# Prevalence of Chronic Back Pain and Associated Factors in Children and Adolescents: Secondary Analysis of the 2001–2019 Health Behavior in School-Aged Children Study

**DOI:** 10.2196/67960

**Published:** 2025-08-06

**Authors:** Camila Miño, Héctor Gutiérrez-Espinoza, Jorge Olivares-Arancibia, Rodrigo Yáñez-Sepúlveda, Daniel Duclos-Bastías, Felipe Araya-Quintanilla, Lee Smith, José Francisco López-Gil

**Affiliations:** 1 School of Medicine, Universidad Espíritu Santo, Av Samborondón 320, Sector La Puntilla, Samborondón, 09-01-952, Ecuador, 593 042835630; 2 Faculty of Education, Universidad Autónoma de Chile, Santiago, Chile; 3 AFySE Group, Research in Physical Activity and School Health, School of Physical Education, Faculty of Education, Universidad de las Américas, Santiago, Chile; 4 Faculty Education and Social Sciences, Universidad Andres Bello, Viña del Mar, Chile; 5 Investigación en Gestión Deportiva y Estudios Olímpicos, School of Physical Education, Pontificia Universidad Católica de Valparaíso, Valparaíso, Chile; 6 Escuela de Kinesiología, Facultad de Ciencias de la Rehabilitación y Calidad de Vida, Universidad San Sebastián, Santiago, Chile; 7 Centre for Health Performance and Wellbeing, Anglia Ruskin University, Cambridge, United Kingdom; 8 Department of Public Health, Faculty of Medicine, Biruni University, Istanbul, Turkey; 9 Vicerrectoría de Investigación y Postgrado, Universidad de Los Lagos, Osorno, Chile

**Keywords:** backache, pediatric population, cross-national trends, socioeconomic status, linear mixed model, cross-sectional study, health outcomes, prevalence, time trends, risk factor, lifestyle, health care access, overweight, obesity

## Abstract

**Background:**

Backache, which is considered a leading cause of adult disability worldwide, is seldom studied in children and adolescents despite it being associated with multiple detrimental health outcomes in this age group.

**Objective:**

The aim of this study is to explore the prevalence, time trends, and correlates of chronic backache among children and adolescents.

**Methods:**

Data were extracted from the cross-sectional Health Behavior in School-Aged Children (HBSC) study, which collected data from 2001 to 2019. The final sample size for this study consisted of 1,011,368 children and adolescents (515,899/1,011,368 or 51.01% were girls). Prevalence estimates and time trends were calculated using weighted proportions. Generalized linear mixed models were conducted to determine whether certain correlates (ie, age group, sex, socioeconomic status, excess weight status, or year of data collection) were associated with higher odds of reporting chronic backache in the population of the HBSC. Additionally, we analyzed country-level changes in chronic backache trends over time.

**Results:**

The weighted global prevalence of chronic backache among children and adolescents was 6.6% (n=12,641; wave 2017-2019). The time trend indicated a general increase in the prevalence of chronic backache in children and adolescents over time (from 2001 to 2019). Indeed, Northern and Eastern Europe, as well as parts of North America, presented relatively higher weighted prevalence rates of chronic backache, ranging from 7% to 9%. This corresponded to approximately 70,796 to 91,023 cases of 1,011,368 participants. Regarding correlates, older age (aged 12.5‐14.5 y: odds ratio [OR] 1.10, 95% confidence interval [CI] 1.05‐1.15; *P*<.001; aged 14.5‐17 y: OR 1.27, 95% CI 1.22‐1.32; *P*<.001), female sex (OR 1.22, 95% CI 1.17‐1.28; *P*<.001), excess weight (OR 1.14, 95% CI 1.11‐1.17; *P*<.001), and later years of data collection (OR 1.04, 95% CI 1.04‐1.05; *P*<.001) were associated with higher odds of reporting chronic backache. In contrast, medium (OR 0.83, 95% CI 0.81‐0.85; *P*<.001) and high (OR 0.90, 95% CI 0.86‐0.93; *P*<.001) socioeconomic status showed lower odds of experiencing chronic backache than low socioeconomic status.

**Conclusions:**

This study revealed a sustained upward trend in the prevalence of backache in children and adolescents over the years (2001‐2019), especially in North America and Northern and Eastern Europe. In addition, older age, female sex, excess weight, and later years of data collection were identified as correlates of experiencing chronic backache. Moreover, middle and high socioeconomic status were associated with lower odds of reporting chronic backache, potentially indicating differences in lifestyle, access to health care, or other protective factors.

## Introduction

Backache, generally characterized by pain and discomfort in the lumbar region, can be categorized based on duration [1]. Almost everyone experiences acute back pain at least once in their lifetime, making it the leading cause of disability worldwide and imposing a significant burden on both individuals and society [2,3]. According to a Global Burden of Disease study, backache affected almost 619 million individuals worldwide in 2020; however, 843 million back pain cases are expected to occur by 2050 [2]. Approximately 5% of children may experience backache at any given moment, with prevalence rates ranging from 1% to 6% in children and between 18% and 51% in adolescents [4,5]. Furthermore, back pain is more common in females, and its occurrence tends to increase with age [2,4]. Backache can diminish the overall perception of health-related quality of life, negatively impacting both physical and emotional well-being [6]. Indeed, it contributes to disability and disrupts daily activities for approximately 10% to 40% of adolescents, representing a critical public health challenge, as it has been associated with a greater probability of chronic backache later in life [6,7].

The development of back pain is influenced by a variety of multifactorial causes and risk factors [8]. Nevertheless, the biopsychosocial model posits that backache arises from interactions among biological elements, such as intervertebral disc degeneration, which are linked to aging, obesity, genetic factors, and physical strain, as well as psychosocial influences, which encompass conditions such as depression, anxiety, and social isolation [3,9]. Most importantly, engagement in competitive sports and older age groups are persistently associated with backache in children and adolescents [9]. Moreover, environmental factors, including socioeconomic status, significantly influence outcomes, as families with more resources may be more likely to offer their children superior nutrition and increased opportunities for engagement in extracurricular activities [10]. Likewise, sedentary behaviors, such as spending more than 15 hours per week on electronic devices, have been associated with backache [7]. Specifically, prolonged screen time often results in poor posture, leading to musculoskeletal strain, muscle spasms, fatigue, and potential intervertebral disc degeneration [3]. These lifestyle habits can weaken muscles, reduce flexibility, impair proprioception, and destabilize the spine, increasing the risk of injury and pain [3,7,11].

The Health Behavior in School-Aged Children (HBSC) study is a collaborative cross-sectional international study conducted by the World Health Organization that has been conducted every 4 years since 1983. This study aims to monitor and enhance the understanding of health, health behaviors, and the contextual factors influencing the lives of children and adolescents [12]. Previous research has reported cross-national trends in back pain in the adolescent population using results from the HBSC study. For example, Roy et al [13] reported a greater prevalence of back pain from 2001‐2002 to 2013‐2014, especially among older girls from the HBSC study. Additionally, chronic back pain has been associated with early menarche in girls, sleep difficulties and psychological symptoms, and increased screen time and obesity in adolescents according to the HBSC study [14-16]. In this context, by understanding how widespread backache is in younger populations, interventions can be implemented early to prevent the condition from worsening or becoming chronic in adulthood. Moreover, since backache is a leading cause of disability, identifying its prevalence in younger populations can help allocate resources, raise awareness, and prioritize public health initiatives aimed at reducing its impact not just in younger populations but across the lifespan. Nevertheless, updated analyses of the prevalence and temporal trends in this population group are scarce. This study aimed to explore the prevalence, time trends, and correlates (ie, age, sex, socioeconomic status, excess weight status, and year of data collection) of chronic backache among children and adolescents from 45 countries between 2001 and 2019.

## Methods

### Study Design and Population

This research extracted data from the 2001‐2002, 2005‐2006, 2009‐2010, 2013‐2014, and 2017‐2019 waves of the HBSC study’s open access repository. In each round of the survey, various nationally representative samples of students aged 10‐17 years participated in an internationally standardized questionnaire administered in school settings. The children and adolescents were chosen randomly from their respective schools to participate in the study and anonymously completed a standardized test administered in their native language. The students had the option to leave questions unanswered. The study sample included children and adolescents aged between 10 and 17 years from different countries: Albania, Armenia, Austria, Azerbaijan, Belgium (both Flanders and Wallonia), Bulgaria, Canada, Croatia, the Czech Republic, Denmark, England, Estonia, Finland, France, Georgia, Germany, Greece, Greenland, Hungary, Iceland, Israel, Italy, Kazakhstan, Latvia, Lithuania, Luxembourg, Malta, the Netherlands, North Macedonia, Norway, Poland, Portugal, the Republic of Moldova, Romania, the Russian Federation, Scotland, Serbia, Slovenia, Spain, Sweden, Ukraine, and Wales [12]. In the 2001‐2002 wave, data were collected from 35 countries, whereas in 2005‐2006, 41 countries participated. Similarly, in 2009‐2010 and 2013‐2014, data were gathered from 40 and 41 countries, respectively. In the most recent wave, 2017‐2019, participation expanded to 45 countries.

After countries and participants with missing data were excluded, the total sample size for this study comprised 1,011,368 children and adolescents. During the 2001‐2002 wave, 162,305 children and adolescents were analyzed, consisting of 78,936 boys (48.6%) and 83,369 girls (51.4%). In the 2005‐2006 wave, the study included 205,938 adolescents, with 101,047 (49.1%) boys and 104,891 (50.9%) girls. For the wave of 2009‐2010, a total of 213,595 children and adolescents were included, with 105,099 (49.2%) boys and 108,496 (50.8%) girls. Similarly, in the 2013‐2014 wave, 214,080 children and adolescents participated, comprising 105,414 (49.2%) boys and 108,666 (50.8%) girls. Finally, in the 2017‐2019 wave, the analysis included 240,951 children and adolescents, with 118,788 (49.3%) boys and 122,163 (50.7%) girls.

### Ethical Considerations

The original data collection was approved by an institutional review board in each participating country, and written informed consent was obtained from schools and adolescents and their parents or legal guardians. As this study involved a secondary analysis of fully anonymized and publicly available data, no additional ethical approval or permission was required. The HBSC survey waves used in this analysis (2001‐2002, 2005‐2006, 2009‐2010, 2013‐2014, and 2017‐2018) are freely accessible for research purposes. Further details on the HBSC study design and procedures are available elsewhere [17,18].

### Procedures

#### Backache

In all waves, participants were asked the following question to report backache frequency: “How often have you had backache in the last six months?” (response options: 1=approximately every day, 2=more than once a week, 3=approximately every week, 4=approximately every month, 5=rarely or never). These scores were subsequently classified into two categories: chronic backache and no chronic backache. Chronic backache was defined as “approximately every day” backache. For further analysis, the data were categorized by wave and country. This involved recording the total number of children and adolescents with chronic backache identified in the waves from 2001‐2002, 2005‐2006, 2009‐2010, 2013‐2014, and 2017‐2019 by country.

#### Sociodemographic and Socioeconomic Data

Sociodemographic data encompassed both sex (males and females) and age categories, specifically, 10‐12.5, 12.5‐14.5, and 14.5‐17 years. The socioeconomic status of children and adolescents was assessed through 4 measures related to family and local socioeconomic position. For instance, in the study waves of 2001-2002, 2005-2006, and 2009-2010, a series of questions were used to assess socioeconomic status, such as “Does your family own a car, van, or truck?” “Do you have your own bedroom for yourself?” “During the past 12 months, how many times did you travel away on holiday with your family?” “How many computers do your family own?” “How well off do you think your family is?” and “Some young people go to school or to bed hungry because there is not enough food at home. How often does this happen to you?” The occupational socioeconomic status of parents was evaluated with various socioeconomic status levels, job-related statuses, and other categories. Additionally, in the 2013-2014 wave, two questions were added: “Does your father/mother have a job?” and “Father/mother occupation socioeconomic status” [17]. Finally, during the 2017-2019 study survey, the Family Affluence Scale-III was used to evaluate socioeconomic status [19]. The scale comprises 6 items and calculates an aggregate index ranging from 0 to 13 points, and each item is scored between 0 and 3 (low socioeconomic status=0‐7 points, medium socioeconomic status=8‐11 points, and high socioeconomic status=12‐13 points). The individual sum scores were converted into proportional ranks, reflecting the relative family affluence of participants within their respective countries. These scores were subsequently classified into 3 categories: the lowest 20%, the middle 60%, and the highest 20% for each country.

#### Anthropometric Variables

Across all waves, children and adolescents provided self-reported data on their body weight and height, from which body mass index (BMI) was derived (kg/m^2^). It should be mentioned, in the 2017‐2019 wave, BMI classification adhered to the standards outlined by the International Obesity Task Force [20] (1=thinness, 2=normal weight, 3=overweight, 4=obesity). For further analysis, excess weight (ie, overweight or obesity) was determined as follows: 0=no excess weight (ie, thinness or normal weight); 1=excess weight (ie, overweight or obesity).

### Statistical Analysis

All the statistical analyses were performed via R statistical software (version 4.4.0; R Core Team) along with RStudio (2024.04.1+748; Posit PBC). For the main analyses, missing data were handled using the listwise deletion method, in which only cases with complete data for all variables included in the model were retained. To adjust for the complex survey design of the HBSC study, we applied the provided survey weights as probability weights. These weights account for unequal probabilities of selection, nonresponse, and poststratification adjustments. For descriptive purposes, the data in this research were expressed in terms of weighted number and percentages. Prevalence estimates and time trends were calculated using weighted proportions. For analytical purposes, a generalized linear mixed model (GLMM) was fitted using the *glmer* function from the *lme4* package to examine the sociodemographic and anthropometric correlates associated with chronic backache. This model included country as a random intercept to account for between-country variability, while age group, sex, socioeconomic status, BMI status, and year of data collection were included as fixed effects. From this model, odds ratios (ORs) and their corresponding 95% confidence intervals (CIs) were computed to determine the strength and precision of these associations. Given the cross-sectional design, ORs from the GLMM are interpreted as measures of association rather than causal inference. The significance of the additional country variance in the multilevel model was determined via the likelihood ratio test. A *P* value below .05 was considered to indicate statistical significance, and 95% CIs are presented. Multicollinearity was assessed using variance inflation factors (VIF), which measure the ratio of the variance of a parameter estimate in a full model to its variance in a single-parameter model.

Interaction terms (age group × sex, socioeconomic status × age group, and socioeconomic status × sex) were tested to assess their contribution to the model. Model performance was evaluated using the Akaike information criterion (AIC), the Bayesian information criterion (BIC), log-likelihood, and deviance. Model selection was based on likelihood ratio tests and AIC/BIC criteria, retaining only interactions that significantly improved model fitness. Random effect variance was also examined to assess the contribution of country-level variability to the model. To mitigate the risk of bias arising from missing data, we performed supplementary analyses using multiple imputation techniques. We used multivariate imputation through chained equations to evaluate the presence of any missing values in the dataset, under the assumption that the missing data occurred at random [21]. The *mice* package was used to replace missing values using chained equations, resulting in the creation of 42 data sets with multiple imputations. This approach adhered to the guideline of setting the number of imputations (m) to exceed 100 times the maximum percentage of missing data [22].

## Results

Table 1 describes the weighted characteristics of the study participants classified by chronic backache and no chronic backache. In general, participants aged 14.5‐17 years had a greater prevalence of chronic backache (23,051/333,419, 6.9%) than did those between 10‐12.5 years (14,865/325,849, 4.6%) and 12.5‐14.5 years (19,415/344,552, 5.6%). Furthermore, more girls had chronic backache (34,348/516,442, 6.7%) than did boys (23,518/494,677, 4.8%). Similarly, 6.4% (17,153/266,985) of children and adolescents with low socioeconomic status had chronic backache, whereas 5.3% (30,286/566,998) and 5.7% (7282/127,488) of those with middle and high socioeconomic status, respectively, had chronic backache. Moreover, 6.2% (11,660/188,106) of participants with excess weight status had chronic backache, whereas 5.5% (35,710/644,951) of individuals with normal weight status reported chronic backache. The weighted prevalence of chronic backache among participants increased from 2001-2002 (7774/159,664, 4.9%) to 2017-2019 (12,641/192,396, 6.6%). The descriptive data of the study participants using a multiple imputation analysis can be found in Multimedia Appendix 1.

**Table 1.** Weighted prevalence of chronic and nonchronic backache across demographic and anthropometric characteristics (Health Behavior in School-Aged Children study 2001‐2019; N=1,011,368).^a^

Variable and category		No chronic backache, n (%)	Chronic backache, n (%)
**Age group (total=1,004,010; missing=7358)**			
	10‐12.5 y	310,984 (95.4)	14,865 (4.6)
	12.5‐14.5 y	325,137 (94.4)	19,415 (5.6)
	14.5‐17 y	310,368 (93.1)	23,051 (6.9)
**Sex (total=1,011,368; missing=0)**			
	Male	471,159 (95.2)	23,518 (4.8)
	Female	482,094 (93.3)	34,348 (6.7)
**Socioeconomic status (total=959,784; missing=51,584)**			
	Low	249,832 (93.6)	17,153 (6.4)
	Medium	536,712 (94.7)	30,286 (5.3)
	High	120,206 (94.3)	7282 (5.7)
**Excess weight status**^**b**^ **(total=831,690; missing=179,678)**			
	No^c^	609,241 (94.5)	35,710 (5.5)
	Yes^d^	176,446 (93.8)	11,660 (6.2)
**Year of data collection (total=799,692; missing=211,676)**			
	2001-2002	151,890 (95.1)	7774 (4.9)
	2005-2006	186,229 (94.5)	10,799 (5.5)
	2009-2010	194,662 (94.3)	11,671 (5.7)
	2013-2014	41,646 (94.8)	2275 (5.2)
	2017-2019	179,755 (93.4)	12,641 (6.6)

a Values are weighted to account for the complex sampling design. Percentages represent the prevalence of chronic backache and no chronic backache within each subgroup.

b According to the cutoff points for body mass index by the International Obesity Task Force [20].

c No excess weight includes participants with thinness or normal weight.

d Excess weight includes participants with overweight or obesity.

**Figure 1.**
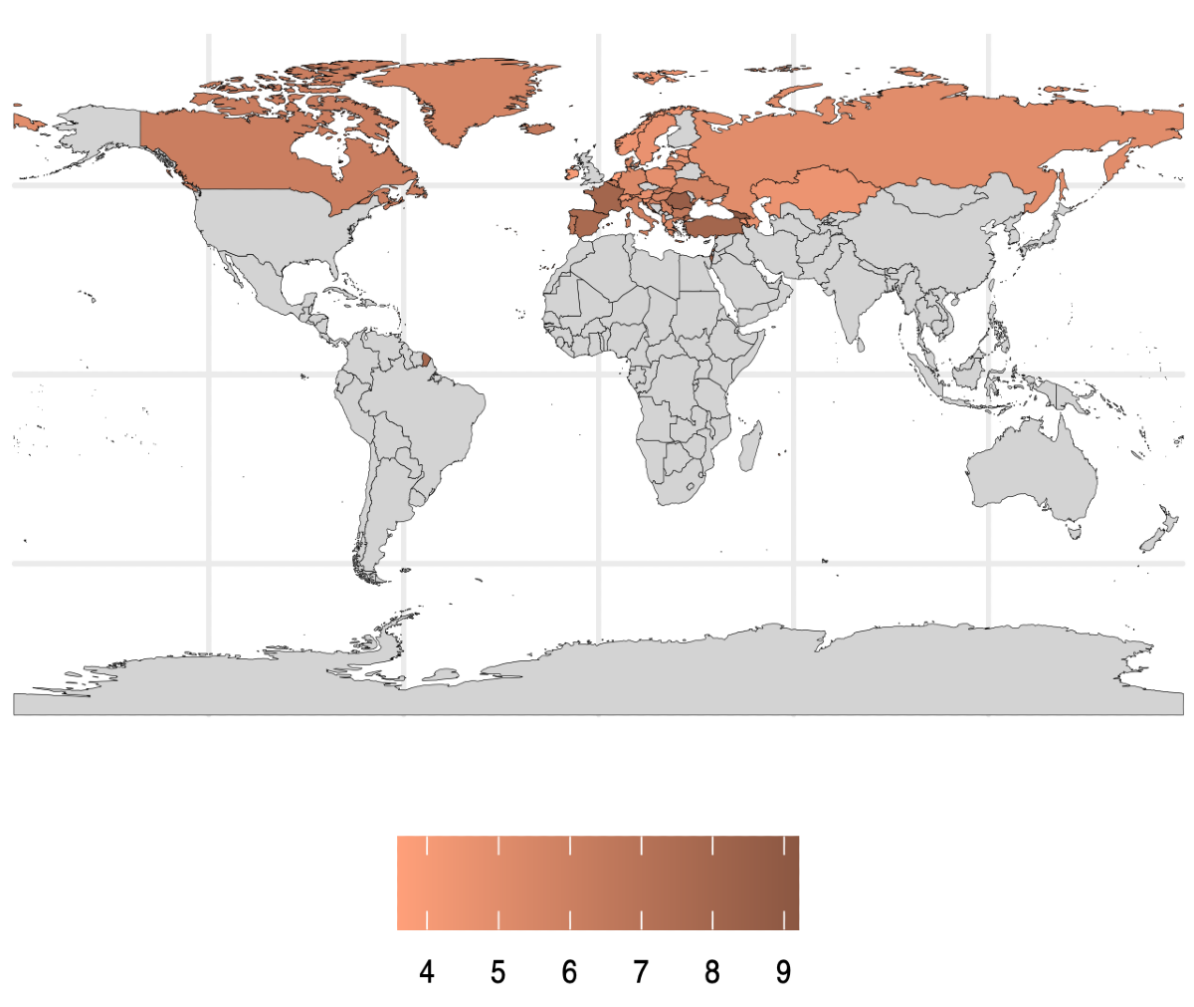
Prevalence of chronic backache in the 10- to 17-year-old population of the Health Behavior in School-Aged Children cross-sectional study (N=1,011,368; 2001‐2019).

Figure 1 illustrates the prevalence of chronic backache among 1,011,368 participants from the HBSC study between 2001 and 2019, with data from multiple countries worldwide. Countries in Northern and Eastern Europe, as well as parts of North America, presented a relatively high prevalence of chronic backache, with values ranging from 7% to 9% (70,796/1,011,368 and 91,023/1,011,368, respectively). In comparison, some parts of Western Europe and a few countries in Asia presented moderate prevalence rates, ranging from approximately 5% to 7% (50,568/1,011,368 and 70,796/1,011,368, respectively). Multimedia Appendix 2 provides the prevalence rates of chronic and no chronic backache by country.

**Figure 2.**
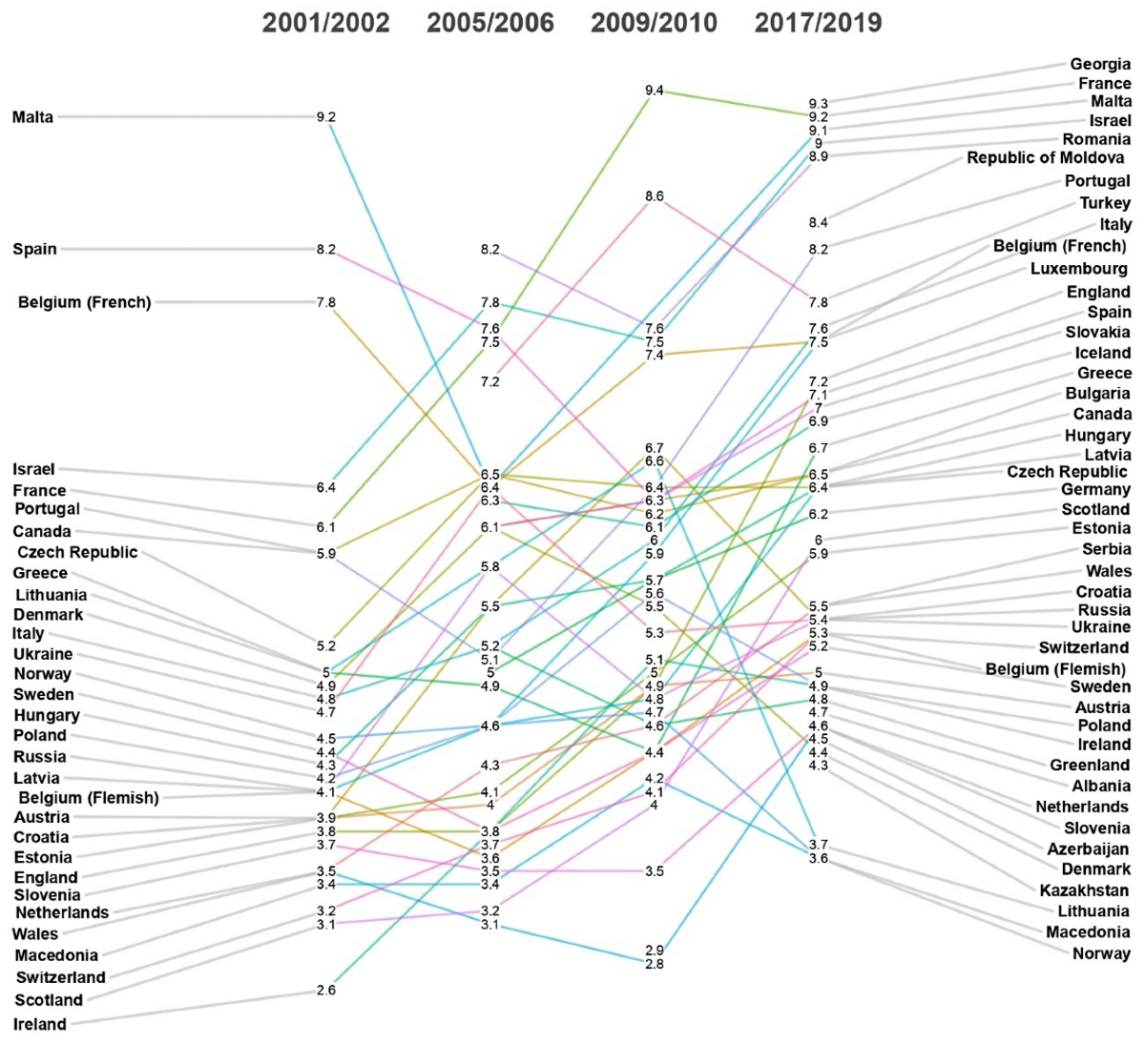
Temporal trends of chronic backache in the 10- to 17-year-old population of the Health Behavior in School-Aged Children cross-sectional study (2001‐2019) stratified by country and wave (N=1,011,368).

Figure 2 displays a detailed breakdown of the temporal trends in chronic backache among children and adolescents across various countries from 2001 to 2019. This trend indicates a general increase in the prevalence of chronic backache in children and adolescents over time, especially from 2001‐2002 to 2017‐2018. In 2017‐2019, the highest prevalence was observed in Georgia (1176/12,641, 9.3%), France (1163/12,641, 9.2%), and Malta (1150/12,641, 9.1%). France and Israel presented significant increases in chronic backache prevalence across the years. Norway (354/12,641, 2.8%), Macedonia (366/12,641, 2.9%), and Lithuania (379/12,641, 3.0%) presented the lowest prevalence rates in 2017-2019. Countries such as Ireland and Switzerland remained consistently low throughout the observed period. Additionally, some countries showed a steady increase (eg, Israel, Romania, Portugal), whereas others had more fluctuations (eg, Belgium, Spain). In comparison, a few countries (eg, Croatia, Poland) remained relatively stable, with slight increases in later years. Multimedia Appendix 3 provides the chronic backache temporal trends stratified by country and wave.

Table 2 presents the results of a GLMM evaluating factors associated with the probability of having chronic backache in the 10- to 17-year-old population of the HBSC study data from 2001‐2019, including the interaction between age group and sex. We tested interaction terms to evaluate their contribution to the model fit. The inclusion of the age group × sex interaction significantly improved the model (*χ*²_2_=85.37; *P*<.001), reducing AIC/BIC values compared to the base model. In contrast, neither the model without any interaction (Multimedia Appendix 4) nor those including age group × socioeconomic status (Multimedia Appendix 5) or sex × socioeconomic status (Multimedia Appendix 6) resulted in a better fit. Therefore, only the age group × sex interaction was retained in the final model. Multicollinearity was checked using VIF, and all variables showed acceptable values (VIF <5), indicating no serious collinearity issues. Compared with participants aged 10.5‐12.5 years, adolescents aged 14.5‐17 years were 27% more likely to experience chronic backache (OR 1.27, 95% CI 1.22‐1.32; *P*<.001), whereas participants aged 12.5‐14.5 years were 10% more likely to experience chronic backache than individuals aged 10‐12.5 years (OR 1.10, 95% CI 1.05‐1.15; *P*<.001). In terms of sex, girls were 22% more likely to experience chronic backache than boys (OR 1.22; 95% CI 1.17‐1.28; *P*<.001). Moreover, middle socioeconomic status was associated with lower odds of chronic backache than low socioeconomic status (OR 0.83, 95% CI 0.81‐0.85; *P*<.001). Similarly, participants with high socioeconomic status had a 10% lower probability of chronic backache (OR 0.90, 95% CI 0.86‐0.93; *P*<.001) than did participants with low socioeconomic status. In addition, children and adolescents with overweight were more likely to experience chronic backache than were those with normal weight (OR 1.14, 95% CI 1.11‐1.17; *P*<.001). Additionally, it was identified that the later the year of data collection, the higher the odds of having chronic backache (OR 1.04, 95% CI 1.04‐1.05; *P*<.001). Multimedia Appendix 7 presents the full GLMM estimating the odds of having chronic backache, including the interaction between age group and sex, based on a multiple imputation analysis. Notably, the results remained consistent regardless of the method used to handle missing data (listwise deletion method versus multiple imputation method). Additionally, the complete results of the model without interaction, as well as models including the interactions between age group and socioeconomic status and between sex and socioeconomic status, based on multiple imputation analysis, are provided in the supplementary material (Multimedia Appendices 8-10, respectively).

**Table 2.** Generalized linear mixed model to evaluate the odds of having chronic backache in the 10- to 17-year-old population of the Health Behavior in School-Aged Children cross-sectional study (2001‐2019), including the interaction between age group and sex.

Predictor	Odds ratio^a^	95% CI	*P* value
**Age group**			
10‐12.5 y	Reference		
12.5‐14.5 y	1.10	1.05-1.15	<.001
14.5‐17 y	1.27	1.22-1.32	<.001
**Sex**			
Male	Reference		
Female	1.22	1.17-1.28	<.001
**Socioeconomic status**			
Low	Reference		
Medium	0.83	0.81-0.85	<.001
High	0.90	0.86-0.93	<.001
**Excess weight status**			
No excess weight	Reference		
Excess weight	1.14	1.11-1.17	<.001
Year of data collection (per one year)	1.04	1.04-1.05	<.001
**Age group × sex**			
12.5‐14.5 y × female	1.19	1.12-1.26	<.001
14.5‐17 y × female	1.31	1.24-1.38	<.001

a Model fit was assessed using the Akaike information criterion (452,439) and the Bayesian information criterion (452,569). The model’s log-likelihood was –226,208, with a deviance of 452416.8. This analysis includes only cases with complete data for all variables in the analysis (n=599,553).

## Discussion

This study presented the prevalence, time trends, and associated factors (age, sex, socioeconomic status, and excess weight) of chronic backache among a large sample of children and adolescents from the HBSC study in 45 countries. Currently, there is limited evidence reporting the epidemiology and associated factors of chronic backache in younger populations using data from the HBSC study from 2001 to 2019. Nevertheless, Roy et al [13] reported increased trends in chronic back pain among females and older adolescents using data from the HBSC study from 2001 to 2014; however, they did not include the related variables explored in our analysis. Other previous analyses used HBSC data to analyze back pain. For example, Roman-Juan et al [14] reported that screen time and obesity were associated with chronic back pain in adolescents in the HBSC study between 2002 and 2014. Similarly, findings from the HBSC study between 2002 and 2018 indicated a general increase of 0.5% in the prevalence of backache in adolescents, which was partially influenced by sleep difficulties and psychological symptoms [15]. Moreover, insights from the HBSC study (2002‐2014) revealed an association between an increase in early menarche and the prevalence of chronic back pain among girls [16].

Overall, our findings indicated a greater prevalence of chronic backache in children and adolescents residing in Northern and Eastern Europe, which can be attributed to a combination of cultural, psychosocial, environmental, health care access, and socioeconomic factors. In fact, the literature highlights that socioeconomic differences between European countries may play a role in the prevalence of back pain among adolescents, with higher prevalence rates observed in countries with less risk of poverty or social exclusion [23,24]. Furthermore, cultural differences in the perception and reporting of pain may play a role in the variation in prevalence rates among European countries [25]. Besides, the colder climate in Northern and Eastern Europe can lead to muscle stiffness and reduced physical activity, which are risk factors for chronic musculoskeletal complaints such as back pain [26,27]. In addition, older age, female sex, excess weight, lower socioeconomic status, and later years of data collection were associated with a greater likelihood of chronic backache. These results are in line with those of previous studies [9,13,23,28-33]. Moreover, the etiology of backache in children and adolescent populations can be attributed to a wide range of medical conditions across various disease categories, regardless of age [34]. This is particularly evident in cases involving infectious diseases, oncological disorders, and congenital anomalies affecting spinal structure [3,34]. However, the specific mechanisms by which nonspecific back pain may develop in children and adolescents are diverse and not fully understood (eg, genetics, hormonal and biological changes, diet, physical activity and sedentary behaviors, psychological factors, and socioenvironmental factors) [3,9,34]. Consequently, it is important to understand the mechanisms by which they may promote back pain in this population group because it may be regarded as a red flag for underlying medical conditions or chronic backache in the future. Despite this, several potential explanations could account for our results.

Adolescence is a period of rapid physical growth; therefore, sudden changes in height can stress the developing spine [3,35]. Research has indicated that the musculoskeletal systems of children insufficiently develop during peak height velocity in puberty to withstand abrupt mechanical load variations in the spine [34,36]. This was attributed to the disparity in growth rates between the legs and the trunk, where the long bones of the legs undergo a growth spurt prior to the shorter bones of the trunk [36]. Likewise, the higher prevalence of back pain in girls than in boys could be due to hormonal and biological changes during puberty [34,35]. The onset of puberty resulted in an increase in height and muscle mass among boys, whereas girls experienced an increase in body fat [35]. Additionally, estrogen and progesterone can influence muscle and joint health, leading to increased vulnerability to pain, as well as early menarche [16,37]. International cross-sectional studies have reported significant differences between boys and girls, with a higher incidence of backache among girls (*P*<.001) [28,30,33].

Obesity is increasingly recognized as a significant public health concern, impacting 1 in 3 individuals below the age of 18 years [38]. Excess weight places additional strain on the lumbar spine and musculoskeletal system, which may be linked to misalignment of vertebrae, discs, and surrounding muscles [37]. Additionally, obesity is associated with chronic inflammation and metabolic changes that can affect musculoskeletal health and are associated with pain [3,38]. Similarly, obesity is often linked to lower levels of physical activity, which could weaken the core muscles that support the spine [39,40]. Despite these possible explanations, the association of excess weight with back pain has been controversial in the literature. Some studies have not identified an association between pediatric backache and physical factors (such as height, weight, or BMI) [9,41]. However, the meta-analysis of García-Moreno et al [42] reported a significant association between overweight-obesity and back pain (OR 1.18, 95% CI 1.14‐1.23) compared with normal weight in children and adolescents.

A growing body of evidence suggests that psychological and psychosocial factors significantly contribute to the etiology of backache [3,7]. Stress has been associated with muscle tension and reduced physical activity and affects pain perception, increasing the prevalence of back pain in this age group [3]. In particular, psychological distress in childhood has been related to a higher likelihood of developing spinal pain in adolescence by 33% (OR 1.33, 95% CI 1.01‐1.76) [11]. Additionally, Batley et al [43] reported that low mood, bad mood, nervousness, difficulty sleeping, and loneliness were related to spinal pain among Danish children and adolescents aged 11‐13 years. In addition, adverse psychological factors are associated with a reduced range of motion in the spine and increased trunk muscle activation in individuals experiencing back pain according to a systematic review and meta-analysis [44]. Adolescents from lower socioeconomic status backgrounds may have reduced access to physical activity opportunities, proper health care, or ergonomic school environments [45]. This lack of resources can result in weaker muscles, reduced flexibility, and poor posture, all of which are related to back pain [3,45]. Hestbaek et al [46] reported that a favorable social background during adolescence served as a protective factor against chronic backache in adulthood, whereas no correlation was found with any form of back pain. Furthermore, Huang et al [47] reported that although most backache cases are resolved acutely, approximately 30% progress to a chronic form. In this context, socioeconomic status is one of the most significant determining factors in the transition to chronic backache compared with traditional biomedical descriptors [47]. In addition, back pain represents an important burden to global public health in younger populations. According to the Global Burden of Disease study, in 2019, the disability-adjusted life year rate of back pain was 627.66 (95% CI 419.71‐866.97) among individuals aged 15‐39 years [48].

The findings in our study have important implications for clinical practice. These results suggest that chronic backache is a widespread complaint in children and adolescents, and its prevalence varies across populations and geographic regions. In addition, the prevalence rates have increased over time, in part due to heightened awareness and detection of chronic pain in children and recognition of its impact. Identification of the factors that contribute to the onset and persistence of back pain in children is important to inform preventive strategies and potential treatments. Although beyond the scope of this study, besides the educational and social aspects, backache in children and adolescents carries economic implications, as it has been associated with increased rates of medical visits.

The present analysis has several limitations that should be considered when the findings are interpreted. Importantly, anthropometric data (weight and height) and backache were self-reported through questionnaires rather than objective methods, which could introduce recall or social desirability bias into the findings. Besides, our study lacks the analysis of national-level covariates (such as the Gini coefficient or the proportion of health care expenditure) to explain regional differences. In addition, the cross-sectional design used in the study limited the capacity to determine a causal relationship between the observed outcomes. Consequently, future prospective cohort observational studies are needed to provide valuable insights into how different exposures, behaviors, and conditions affect backache in children and adolescent populations over time, helping to identify preventive measures, high-risk groups, and the effectiveness of existing interventions. A significant advantage of the study lies in its utilization of a substantial and representative sample of children and adolescents across 45 countries, which consequently strengthens the external validity of the results. Furthermore, we performed a multiple imputation analysis to assess the robustness and enhance the generalizability of our results.

This study revealed a sustained upward trend in the prevalence of chronic backache in children and adolescents over the years (2001‐2019), especially in North America and Northern and Eastern Europe. In addition, older age, female sex, excess weight, and later years of data collection were identified as correlates of experiencing chronic backache. Additionally, a higher socioeconomic status was associated with a lower likelihood of chronic backache, potentially indicating differences in lifestyle, access to health care, or other protective factors. Given that backache is a primary contributor to disability worldwide, assessing its prevalence among younger demographics is important for identifying potential correlates and informing prevention or intervention strategies specifically designed for younger populations. Nevertheless, the causal nature of these associations remains unclear and warrants further prospective research to elucidate potential mechanisms. Moreover, unmeasured factors may also contribute to the prevalence of chronic backache among children and adolescents.

## Supplementary material

10.2196/67960 Multimedia Appendix 1 Table S1. Descriptive data of the study participants using a multiple imputation analysis (N=1,036,869).

10.2196/67960 Multimedia Appendix 2 Table S2. Prevalence rates of chronic and no chronic backache by country in the 10- to 17-year-old population of the Health Behavior in School-Aged Children study (2001-2019).

10.2196/67960 Multimedia Appendix 3 Table S3. Chronic backache temporal trend rates in the 10- to 17-year-old population of the Health Behavior in School-Aged Children study (2001-2019) stratified by country and wave.

10.2196/67960 Multimedia Appendix 4 Table S4. Generalized linear mixed model to evaluate the probability of having chronic backache in the 10- to 17-year-old population of the Health Behavior in School-Aged Children study (2001-2019).

10.2196/67960 Multimedia Appendix 5 Table S5. Generalized linear mixed model to evaluate the probability of having chronic backache in the 10- to 17-year-old population of the Health Behavior in School-Aged Children study (2001-2019), including the interaction between age group and socioeconomic status.

10.2196/67960 Multimedia Appendix 6 Table S6. Generalized linear mixed model to evaluate the probability of having chronic backache in the 10- to 17-year-old population of the Health Behavior in School-Aged Children study (2001-2019), including the interaction between sex and socioeconomic status.

10.2196/67960 Multimedia Appendix 7 Table S7. Generalized linear mixed model evaluating the probability of having chronic backache among 10- to 17-year-olds in the Health Behavior in School-Aged Children cross-sectional study (2001-2019), including an interaction between age group and sex. The model was estimated using multiple imputation.

10.2196/67960 Multimedia Appendix 8 Table S8. Generalized linear mixed model to evaluate the probability of having chronic backache in the 10- to 17-year-old population of the Health Behavior in School-Aged Children study (2001-2019). The model was estimated using multiple imputation.

10.2196/67960 Multimedia Appendix 9 Table S9. Generalized linear mixed model evaluating the probability of having chronic backache among 10- to 17-year-olds in the Health Behavior in School-Aged Children cross-sectional study (2001-2019), including an interaction between age group and socioeconomic status. The model was estimated using multiple imputation.

10.2196/67960 Multimedia Appendix 10 Table S10. Generalized linear mixed model evaluating the probability of having chronic backache among 10- to 17-year-olds in the Health Behavior in School-Aged Children cross-sectional study (2001-2019), including an interaction between sex and socioeconomic status. The model was estimated using multiple imputation.

## Abbreviations

AIC: Akaike information criterion
BIC: Bayesian information criterion
BMI: body mass index
GLMM: generalized linear mixed model
HBSC: Health Behavior in School-Aged Children
OR: odds ratio
VIF: variance inflation factors
