# Supplementary material for: Prevalence of Chronic Back Pain and Associated Factors in Children and Adolescents: Secondary Analysis of the 2001–2019 Health Behavior in School-Aged Children Study
Source: JMIR Public Health Surveill. 2025 Aug 6;11:e67960. doi: 10.2196/67960 (PMC12327913; doi:10.2196/67960)
Supplement: Multimedia Appendix 1 [file publichealth-v11-e67960-s001.docx]

Table S1. Descriptive data of the study participants using a multiple imputation analysis (N = 1,036,869).

| **Variable** | **n (%)** |
| --- | --- |
| Age group |  |
| 10 to 12.5 | 339,969 (32.8%) |
| 12.5 to 14.5 | 356,472 (34.4%) |
| 14.5 to 17 | 340,428 (32.8%) |
| Sex |  |
| Boys | 509,284 (49.1%) |
| Girls | 527,585 (50.9%) |
| Socioeconomic status |  |
| Low | 291,362 (28.1%) |
| Medium | 606,505 (58.5%) |
| High | 139,002 (13.4%) |
| Excess weight status ^a^ |  |
| No excess weight ^b^ | 800,266 (77.2%) |
| Excess weight ^c^ | 236,603 (22.8%) |
| Year |  |
| 2001/2002 | 207,052 (20.0%) |
| 2005/2006 | 13,267 (25.4%) |
| 2009/2011 | 259,859 (25.10%) |
| 2017/2019 | 306,785 (29.5%) |
| ^a^ According to the cutoff points for body mass index by the International Obesity Task Force [20].  ^b^ No excess weight includes participants with thinness or normal weight.  ^c^ Excess weight includes participants with overweight or obesity | |
