# Supplementary material for: Prevalence of Chronic Back Pain and Associated Factors in Children and Adolescents: Secondary Analysis of the 2001–2019 Health Behavior in School-Aged Children Study
Source: JMIR Public Health Surveill. 2025 Aug 6;11:e67960. doi: 10.2196/67960 (PMC12327913; doi:10.2196/67960)
Supplement: Multimedia Appendix 2 [file publichealth-v11-e67960-s002.docx]

**Table S2.** Prevalence rates of chronic and no chronic backache by country in the 10- to 17-year-old population of the Health Behavior in School-Aged Children (2001–2019).

| **Country ^a^** | **No chronic backache**  **n (%)** | **Chronic backache**  **n (%)** |
| --- | --- | --- |
| Albania | 1626 (95.2) | 82 (4.8) |
| Azerbaijan | 4209 (95.3) | 206 (4.7) |
| Austria | 17,407 (95.3) | 853 (4.7) |
| Armenia | 6342 (95.8) | 281 (4.2) |
| Belgium (Flemish) | 18,005 (95.5) | 847 (4.5) |
| Belgium (French) | 16,409 (92.3) | 1366 (7.7) |
| Bulgaria | 8744 (93.5) | 606 (6.5) |
| Canada | 35,854 (93.9) | 2315 (6.1) |
| Croatia | 19,251 (94.4) | 1141 (5.6) |
| Czech Republic | 23,546 (93.7) | 1576 (6.3) |
| Denmark | 16,757 (94.6) | 952 (5.4) |
| Estonia | 16,488 (95.2) | 823 (4.8) |
| France | 27,550 (92.0) | 2395 (8.0) |
| Georgia | 3542 (90.8) | 358 (9.2) |
| Germany | 20,923 (94.7) | 1170 (5.3) |
| Greece | 15,348 (94.7) | 861 (5.3) |
| Greenland | 3865 (94.4) | 230 (5.6) |
| Hungary | 15,241 (94.5) | 892 (5.5) |
| Iceland | 25,477 (93.5) | 1784 (6.5) |
| Ireland | 15,491 (96.0) | 648 (4.0) |
| Israel | 20,293 (91.6) | 1866 (8.4) |
| Italy | 16,173 (94.1) | 1020 (5.9) |
| Kazakhstan | 4341 (95.5) | 203 (4.5) |
| Latvia | 15,250 (95.0) | 811 (5.0) |
| Lithuania | 19,048 (94.7) | 1058 (5.3) |
| Luxembourg | 11,692 (94.0) | 740 (6.0) |
| Malta | 5276 (91.4) | 499 (8.6) |
| Republic of Moldova | 4174 (91.6) | 382 (8.4) |
| Netherlands | 16,990 (96.4) | 628 (3.6) |
| Norway | 16,102 (95.6) | 743 (4.4) |
| Poland | 20,138 (95.2) | 1010 (4.8) |
| Portugal | 15,655 (93.3) | 1133 (6.7) |
| Romania | 12,982 (91.4) | 1221 (8.6) |
| Russia | 23,883 (95.0) | 1257 (5.0) |
| Serbia | 3594 (94.3) | 219 (5.7) |
| Slovakia | 12,580 (93.2) | 914 (6.8) |
| Slovenia | 19,274 (96.1) | 789 (3.9) |
| Spain | 21,965 (92.3) | 1828 (7.7) |
| Sweden | 17,842 (95.4) | 868 (4.6) |
| Switzerland | 22,175 (95.8) | 978 (4.2) |
| Turkey | 15,275 (92.0) | 1337 (8.0) |
| Ukraine | 19,258 (94.3) | 1168 (5.7) |
| Macedonia | 16,691 (96.3) | 645 (3.7) |
| England | 16,522 (96.0) | 691 (4.0) |
| Scotland | 21,246 (96.2) | 845 (3.8) |
| Wales | 27,649 (95.2) | 1403 (4.8) |
| **Total** | 728,144 (94.3) | 43,643 (5.7) |

^a^ Only participants with full data.
