# Supplementary material for: Prevalence of Chronic Back Pain and Associated Factors in Children and Adolescents: Secondary Analysis of the 2001–2019 Health Behavior in School-Aged Children Study
Source: JMIR Public Health Surveill. 2025 Aug 6;11:e67960. doi: 10.2196/67960 (PMC12327913; doi:10.2196/67960)
Supplement: Multimedia Appendix 3 [file publichealth-v11-e67960-s003.docx]

**Table S3.** Chronic backache temporal trend rates in the 10- to 17-year-old population of the Health Behavior in School-Aged Children (2001-2019) stratified by country and wave.

| **Country (%)** | **Wave** | | | |
| --- | --- | --- | --- | --- |
|  | **2001/2002**  **(n=7,774)** | **2005/2006**  **(n=10,799)** | **2009/2010**  **(n=11,671)** | **2017/2019**  **(n=12,641)** |
| Albania |  |  |  | 4.7 |
| Armenia |  |  | 2.9 |  |
| Austria | 3.9 | 4.0 | 4.9 | 5.0 |
| Azerbaijan |  |  |  | 4.5 |
| Belgium (Flemish) | 4.1 | 3.6 | 4.4 | 5.3 |
| Belgium (French) | 7.8 | 6.4 | 7.4 | 7.5 |
| Bulgaria |  | 6.1 |  | 6.5 |
| Canada | 5.9 | 6.5 | 6.2 | 6.5 |
| Croatia | 3.9 | 5.5 | 6.7 | 5.4 |
| Czech Republic | 5.2 | 6.5 | 6.4 | 6.4 |
| Denmark | 4.9 | 6.1 | 5.5 | 4.4 |
| Estonia | 3.9 | 4.1 | 5.0 | 5.9 |
| France | 6.1 | 7.5 | 9.4 | 9.2 |
| Georgia |  |  |  | 9.3 |
| Germany |  | 5.0 | 5.7 | 6.2 |
| Greece | 5.0 | 4.9 | 4.4 | 6.7 |
| Greenland |  | 5.2 | 4.6 | 4.8 |
| Hungary | 4.3 | 5.5 | 5.7 | 6.4 |
| Iceland |  | 6.3 | 6.1 | 6.9 |
| Ireland | 2.6 | 3.8 | 5.1 | 4.9 |
| Israel | 6.4 | 7.8 | 7.5 | 9.0 |
| Italy | 4.8 | 5.2 | 6.0 | 7.6 |
| Kazakhstan |  |  |  | 4.3 |
| Latvia | 4.1 | 4.6 | 4.8 | 6.4 |
| Lithuania | 5.0 | 5.8 | 6.6 | 3.7 |
| Luxembourg |  | 4.6 | 5.9 | 7.5 |
| Malta | 9.2 | 6.4 |  | 9.1 |
| Republic of Moldova |  |  |  | 8.4 |
| Netherlands | 3.5 | 3.1 | 2.8 | 4.6 |
| Norway | 4.5 |  | 4.7 | 3.6 |
| Poland | 4.2 | 4.6 | 5.6 | 4.9 |
| Portugal | 5.9 | 5.1 | 6.4 | 8.2 |
| Romania |  | 8.2 | 7.6 | 8.9 |
| Russia | 4.1 | 5.8 | 4.8 | 5.4 |
| Serbia |  |  |  | 5.5 |
| Slovakia |  | 6.1 | 6.3 | 7.0 |
| Slovenia | 3.7 | 3.5 | 3.5 | 4.6 |
| Spain | 8.2 | 7.6 | 6.3 | 7.1 |
| Sweden | 4.4 | 3.8 | 4.4 | 5.2 |
| Switzerland | 3.2 | 3.7 | 4.1 | 5.3 |
| Turkey |  | 7.2 | 8.6 | 7.8 |
| Ukraine | 4.7 | 6.4 | 5.3 | 5.4 |
| Macedonia | 3.4 | 3.4 | 4.2 | 3.6 |
| England | 3.8 | 3.8 | 4.9 | 7.2 |
| Scotland | 3.1 | 3.2 | 4.0 | 6.0 |
| Wales | 3.5 | 4.3 | 4.6 | 5.5 |
