# Supplementary material for: Prevalence of Chronic Back Pain and Associated Factors in Children and Adolescents: Secondary Analysis of the 2001–2019 Health Behavior in School-Aged Children Study
Source: JMIR Public Health Surveill. 2025 Aug 6;11:e67960. doi: 10.2196/67960 (PMC12327913; doi:10.2196/67960)
Supplement: Multimedia Appendix 4 [file publichealth-v11-e67960-s004.docx]

**Table S4.** Generalized linear mixed model to evaluate the probability of having chronic backache in the 10- to 17-year-old population of the Health Behavior in School-Aged Children (2001–2019).

| **Characteristic** | **OR ^a^** | **95% CI** | ***P*-value** |
| --- | --- | --- | --- |
| Age group |  |  |  |
| 10–12.5 y | Reference |  |  |
| 12.5–14.5 y | 1.21 | 1.18, 1.25 | <.001 |
| 14.5–17 y | 1.48 | 1.44, 1.53 | <.001 |
| Sex |  |  |  |
| Boys | Reference |  |  |
| Girls | 1.45 | 1.42, 1.48 | <.001 |
| Socioeconomic status |  |  |  |
| Low | Reference |  |  |
| Medium | 0.83 | 0.81, 0.85 | <.001 |
| High | 0.90 | 0.86, 0.93 | <.001 |
| Excess weight status |  |  |  |
| No excess weight | Reference |  |  |
| Excess weight | 1.14 | 1.11, 1.17 | <.001 |
| Year of data collection (per one year) | 1.04 | 1.04, 1.05 | <.001 |
| CI, confidence interval; OR, odds ratio.  ^a^ Model fit was assessed using the Akaike Information Criterion (AIC = 253,901) and the Bayesian Information Criterion (BIC = 254,003). The model’s log-likelihood was -126,942, with a deviance of 253,883. This analysis includes only cases with complete data for all variables in the analysis (n = 599,553). | | | |
