# Supplementary material for: Prevalence of Chronic Back Pain and Associated Factors in Children and Adolescents: Secondary Analysis of the 2001–2019 Health Behavior in School-Aged Children Study
Source: JMIR Public Health Surveill. 2025 Aug 6;11:e67960. doi: 10.2196/67960 (PMC12327913; doi:10.2196/67960)
Supplement: Multimedia Appendix 7 [file publichealth-v11-e67960-s007.docx]

Table S7. Generalized linear mixed model evaluating the probability of having chronic backache among 10- to 17-year-olds in the Health Behavior in School-Aged Children (HBSC) cross-sectional study (2001–2019), including an interaction between age group and sex. The model was estimated using multiple imputation.

| **Predictor** | **OR ^a^** | **95% CI** | ***P-*value** |
| --- | --- | --- | --- |
| Age group |  |  |  |
| 10 to 12.5 y | Reference |  |  |
| 12.5 to 14.5 y | 1.09 | 1.05, 1.12 | <.001 |
| 14.5 to 17 y | 1.26 | 1.22, 1.30 | <.001 |
| Sex |  |  |  |
| Boys | Reference |  |  |
| Girls | 1.17 | 1.14, 1.21 | <.001 |
| Socioeconomic status |  |  |  |
| Low | Reference |  |  |
| Medium | 0.83 | 0.82, 0.85 | <.001 |
| High | 0.90 | 0.87, 0.92 | <.001 |
| Excess weight status |  |  |  |
| No excess weight | Reference |  |  |
| Excess weight | 1.12 | 1.09, 1.14 | <.001 |
| Year of data collection (per one year) | 1.03 | 1.03, 1.04 | <.001 |
| Age group × Sex |  |  |  |
| 12.5 to 14.5 × Girls | 1.18 | 1.13, 1.23 | <.001 |
| 14.5 to 17 × Girls | 1.35 | 1.30, 1.41 | <.001 |

CI, confidence interval; OR, odds ratio.

^a^ Model fit was assessed using the Akaike Information Criterion (AIC = 452,276) and the Bayesian Information Criterion (BIC = 452,406). The model’s log-likelihood was -226,127, with a deviance of 452,254. This analysis includes only cases with complete data for all variables in the analysis (*N* = 1,036,869).
