# Supplementary material for: Prevalence of Chronic Back Pain and Associated Factors in Children and Adolescents: Secondary Analysis of the 2001–2019 Health Behavior in School-Aged Children Study
Source: JMIR Public Health Surveill. 2025 Aug 6;11:e67960. doi: 10.2196/67960 (PMC12327913; doi:10.2196/67960)
Supplement: Multimedia Appendix 9 [file publichealth-v11-e67960-s009.docx]

Table S9. Generalized linear mixed model evaluating the probability of having chronic backache among 10- to 17-year-olds in the Health Behavior in School-Aged Children (HBSC) cross-sectional study (2001–2019), including an interaction between age group and socioeconomic status. The model was estimated using multiple imputation.

| **Predictor** | **OR ^a^** | **95% CI** | ***P-*value** |
| --- | --- | --- | --- |
| Age group |  |  |  |
| 10 to 12.5 y | Reference |  |  |
| 12.5 to 14.5 y | 1.20 | 1.16, 1.25 | <.001 |
| 14.5 to 17 y | 1.49 | 1.44, 1.54 | <.001 |
| Sex |  |  |  |
| Boys | Reference |  |  |
| Girls | 1.39 | 1.37, 1.42 | <.001 |
| SES |  |  |  |
| Low | Reference |  |  |
| Medium | 0.84 | 0.81, 0.87 | <.001 |
| High | 0.86 | 0.82, 0.90 | <.001 |
| Excess weight status |  |  |  |
| No excess weight | Reference |  |  |
| Excess weight | 1.12 | 1.09, 1.14 | <.001 |
| Year of data collection (per one year) | 1.03 | 1.03, 1.04 | <.001 |
| Age group × Socioeconomic status |  |  |  |
| 12.5 to 14.5 × Medium | 0.97 | 0.93, 1.02 | .270 |
| 14.5 to 17 × Medium | 1.01 | 0.96, 1.05 | .750 |
| 12.5 to 14.5 × High | 1.07 | 1.00, 1.14 | .042 |
| 14.5 to 17 × High | 1.05 | 0.99, 1.12 | .113 |

CI, confidence interval; OR, odds ratio; SES, Socioeconomic status

^a^ Model fit was assessed using the Akaike Information Criterion (AIC = 452,470) and the Bayesian Information Criterion (BIC = 452,625). The model’s log-likelihood was – 226,222, with a deviance of 452,444. This analysis includes only cases with complete data for all variables in the analysis (*N* = 1,036,869).
